# Physiological changes of cortisol and oxytocin following manual therapy: a scoping review

**DOI:** 10.3389/fresc.2026.1719735

**Published:** 2026-03-05

**Authors:** Andy T. Cao, Murdi S. Alanazi, Rebecca Billings, William R. Reed

**Affiliations:** 1 Rehabilitation Science Program, University of Alabama at Birmingham, Birmingham, AL, United States; 2 Department of Physical Therapy and Health Rehabilitation, College of Applied Medical Sciences, Jouf University, Sakaka, Saudi Arabia; 3 University of Alabama at Birmingham Libraries, Birmingham, AL, United States; 4 Department of Physical Therapy, University of Alabama at Birmingham, Birmingham, AL, United States

**Keywords:** cortisol, manual therapy, massage, oxytocin, scoping review, spinal manipulation, spinal mobilization

## Abstract

**Introduction:**

Neuroendocrine responses to manual therapy (MT), including changes in cortisol and oxytocin, have long been proposed as potential physiological mechanisms of MT-related effects. Over the past three decades, a growing body of literature has examined hormonal/neuropeptide responses following MT; however, findings remain heterogeneous and unevenly distributed across outcomes and MT intervention types. The objective of this scoping review was to map and characterize the existing evidence for changes in cortisol (CORT) and/or oxytocin (OXT) levels in response to three commonly used MT interventions and to identify patterns and gaps within the literature.

**Methods:**

Five databases [Cumulated Index to Nursing and Allied Health Literature (CINAHL), Embase, PubMed, Scopus, and Web of Science] were searched from inception to July 2025. Eligible studies included those which were (a) in English, (b) in humans, (c) original data studies, (d) included a form of MT (massage, osteopathic manipulative treatment, spinal manipulation, etc.), and (e) at least one physiological measure (e.g., saliva, urine, plasma) of CORT and/or OXT. Two reviewers independently screened studies and extracted data using a structured approach consistent with scoping review methodology.

**Results:**

The literature search identified 2,374 studies, with 72 articles meeting the eligibility criteria. Studies varied widely in MT intervention type, population characteristics, sample size, rigor, biological sampling methods, and timing of outcome assessment. Most of the included studies measured cortisol outcomes, whereas fewer investigated oxytocin. Both reported hormonal/neuropeptide responses as mixed across MT intervention approaches.

**Conclusions:**

CORT and OXT responses to MT were variable across studies, reflecting heterogeneity in intervention delivery, sample size, timing of hormone/neuropeptide collection, and analytical methods. This review underscores the need to standardize how MT-related neuroendocrine outcomes are collected, address the many gaps in the evidence particularly as it relates to OXT, and to clarify the potential mechanistic role of CORT and OXT in MT-related clinical outcomes.

**Systematic Review Registration:**

doi: 10.17605/OSF.IO/A78TU, Open Science Framework.

## 1 Introduction

Manual therapy (MT) is a commonly used non-pharmacological passive intervention applied by clinicians and therapists to relieve musculoskeletal pain, restore optimal movement and physiological function in targeted musculoskeletal tissues (1). The American College of Physicians along with several other clinical practice guidelines recommend nonpharmacological MT as a conservative first line of treatment for musculoskeletal pain (2–4). Common MT techniques include massage, joint or spinal mobilization/manipulation, and myofascial release. Beyond its biomechanical and soft tissue effects, MT has been long argued to modulate certain hormones and neuropeptides (e.g., cortisol, oxytocin, etc.) as well as other neuroimmune signaling pathways, including inflammatory mediators and cytokines due to the inherent nature of mechanical touch involved, the resultant soft tissue relaxation, and musculoskeletal pain reduction associated with MT treatment (5–8).

Within the MT literature, neuroendocrine-related outcomes have often focused on cortisol (CORT) and/or oxytocin (OXT); albeit oxytocin has been examined much less despite being closely associated with touch-based interventions. However, the high heterogeneity in MT study design, biological sampling methods, MT treatment parameters, and timing of neuroendocrine physiological release/response and outcome measurement have resulted in mixed findings with the inability to draw definitive conclusions regarding potential mechanistic roles or neuroendocrine-related contributions to clinical efficacy of MT. Therefore, a scoping review approach which aims to describe the extent and characteristics of the available CORT/OXT evidence and identify gaps in knowledge is warranted to serve as an update of evidence existing within the MT literature and a resource for future MT-related neuroendocrine investigations with goals of achieving more definitive conclusions as to potential neuroendocrine-related mechanisms involved in MT.

CORT is an end product of the hypothalamic-pituitary-adrenal axis (HPA-axis) that is involved in regulating the stress response and is an essential mediator between psychological states and health-related outcomes (9). Increased stress and disrupted sleep patterns have been implicated in driving systemic inflammation via upregulating the sympathetic nervous system and HPA-axis activity (i.e., disrupting cortisol levels and receptor sensitivity) which carries the potential of sensitizing peripheral and central neurons resulting in hyperalgesia (10, 11). The relationship between acute and/or chronic pain and dysregulation of the HPA-axis has been well documented in the literature (12–15). CORT plays a critical role in analgesia as it is released during the initial stages of the stress response and functions to suppress inflammation, edema, and reduce pain. More specifically, it decreases local edema and discomfort by inhibiting the early stages of the inflammatory cascade (16). Therefore, any MT interventions that act to reduce stress and/or systemic inflammation biomarkers may reflect broader effects of MT on stress regulation and/or peripheral/central pain-processing.

Multiple reviews investigating changes of various biomarkers have reported low or moderate quality of evidence that spinal manipulation results in immediate CORT level changes (8, 19, 20). However, it is important to note that a majority of these reviewed CORT studies involved healthy (asymptomatic) participants, studies with small sample sizes, and/or presented high heterogeneity that prevented pooling of the data. In a 2021 systematic review and meta-analysis of neuroimmune responses in animals and humans with neuromuscular conditions (7), three studies (*n* = 140 patients) assessed the change in CORT in people with neck and/or back pain following joint mobilization/manipulation treatment and reported increased serum CORT in those with acute back pain (17), increased salivary CORT in those with chronic neck pain (18), and no significant CORT changes in individuals with acute neck pain receiving spinal manipulation compared with sham treatment (16). Data from these three studies could not be pooled because of high heterogeneity in methodology and/or MT interventions, yet they provide sufficient motivation to continue a MT-related line of neuroendocrine investigation.

For massage, a 2011 comprehensive quantitative review of 18 articles (704 individuals) pertaining to massage therapy effects on CORT levels (blood, saliva, urine) reported that massage effects on CORT levels were indeed very small and nonsignificant in the vast majority of studies with a lone exception being a multiple dose effect in children (19). The authors of this review noted at the time that their findings refuted the common assertion that massage significantly reduces cortisol, but they were quick to note that these review findings did not negate massage's sizable and proven reductions of trait anxiety, depression, and certain types of pain, but that mechanisms other than CORT were most likely responsible (19). Despite the findings of this older review, three narrative reviews on massage (19–21), mentioned changes in CORT as a potential physiological mechanism of massage therapy and several recent investigations into changes in CORT levels related to MT interventions reported positive findings warranting further investigation.

In addition to CORT, OXT is a neuropeptide that functions both as a neurotransmitter within the brain and as a hormone released into the bloodstream from the posterior pituitary (neurohypophysis) (22, 23). OXT is synthesized primarily in magnocellular neurons of the bilateral supraoptic (SON) and paraventricular nuclei (PVN) of the hypothalamus and is released into the bloodstream via the posterior pituitary. Of note, both OXT and corticotropin-releasing hormone are produced in the PVN, where reciprocal interactions allow OXT signaling to influence stress-related HPA-axis activity, including attenuation of CORT release under certain conditions (24). OXT also interacts with many other neuropeptides including but not limited to angiotension IV, cholecystokinin octapeptide, opiods, orexin, and leptin, as well as other neurotransmitters such as serotonin and dopamine (24). While OXT primary effects are mediated in the brain, parvocellular OXT neurons of the PVN project extensively toward the brainstem and spinal cord where they form synaptic contacts with neurons involved in autonomic function, pain and analgesia (25, 26). Inhibition of paravocellular OXT neurons in the PVN projecting toward the spinal cord decreased pain thresholds thus increasing pain sensitivity, while activation of these neurons repressed nociception and promoted analgesia. Among peripheral organs with OXT receptor expression and binding are the renal cortex, heart, nociceptive dorsal root ganglion (DRG) neurons, adipocytes and adrenal medulla cells (24). The expression of OXT and its receptor has also been demonstrated in fibroblasts and keratinocytes of human skin (27). It is the nociceptive DRG, connective tissue, and skin-related expression that is of particular interest in potential OXT mechanisms related to MT interventions as evidence for OXT's role in peripheral inflammatory processes is increasing. Among its centrally-mediated behavioral and physiological effects, OXT is released during labor, breastfeeding, sexual activity, physical touch, maternal interaction and bonding, as well as during social interactions (anxiety, trust, sociability) between adults and/or animals (24, 28, 29); but it also serves as a neuromodulator with anxiolytic and analgesic properties (30, 31). OXT release has been associated with cutaneous stimulation such as gentle touch, stroking, massage, and exposure to warmth (32, 33). Given its established involvement in touch-mediated and affiliative processes, MT has been hypothesized to influence OXT release through sustained physical contact and related sensory input inherent to MT-related interventions. However, compared to CORT, substantially fewer MT studies have examined OXT responses, and existing findings remain very limited and mixed thereby warranting additional investigation.

While the purpose of this MT-related scoping review is not to provide a theoretical or physiological mechanistic explanation, broader physiological evidence supports the plausibility of neuroendocrine involvement in clinical MT responses. Various techniques of MT have been shown to influence autonomic nervous system activity, inflammatory mediators, and neuroimmune signaling pathways, all of which are closely linked to regulation of the HPA-axis and OXT signaling (5, 7, 8, 34). Within this broader physiological context, CORT and OXT are integrative neuroendocrine mediators that are influenced by autonomic nervous system activity, inflammatory processes, and neuroimmune signaling pathways involved in stress regulation and pain modulation. Framing these hormones within the established manual therapy evidence base therefore provides a clearer physiological context for their continued investigation to arrive at definitive conclusions. Differences in MT intervention type, treatment duration, study population, sampling methods and varied timing of hormone measurement have resulted in substantial heterogeneity across studies. This high variability across different MT techniques complicates interpretation of findings across studies highlighting the need to better describe, characterize and standardize how the data is collected, where research activity is concentrated, and where neuroendocrine MT-related gaps remain. A strength of this scoping review is to focus on three distinct techniques of MT and not just one MT approach (35–38) to provide a more complete picture of MT-related CORT and OXT studies and evidence.

## 2 Methods

### 2.1 Design

This scoping review was performed following the framework and guidelines of Arksey and O'Malley, with reporting guided by the Preferred Reporting Items for Systematic Reviews and Meta-Analyses extension for scoping reviews, along with more contemporary methodological guidance by Mak and Thomas and the Joanna Briggs Institute Scoping Reviews Methodology Group (36, 38–41). The review process followed the core stages outlined in the Arksey and O'Malley and others, including identification of the research question, identification and selection of relevant studies, data charting, and collation and synthesis of findings (35, 36, 39, 42). MT interventions were defined according to Bearne and Hurley as “a skilled application of passive movement to a joint [and/or muscles] either within (‘mobilization’) or beyond its active range of movement (‘manipulation’)” (43), allowing inclusion of diverse techniques such as spinal manipulation, massage, Anma massage, rib raising, suboccipital fascial release, among others.

### 2.2 Search strategy

**Figure 1:**
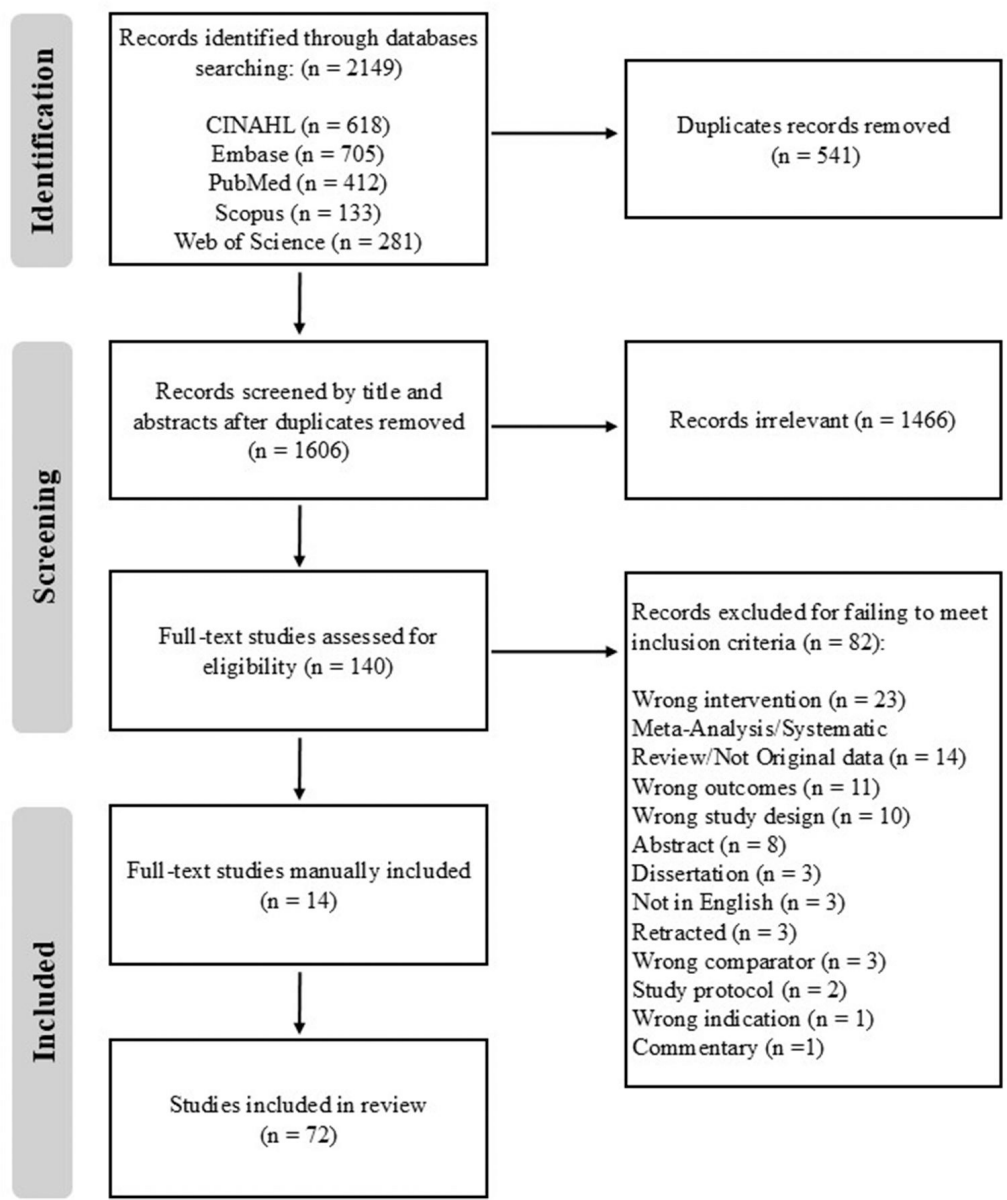
The PRISMA flow chart outlining the study selection process.

A reproducible search strategy was developed from inception through July 2025, in collaboration with a research librarian (RB), and was applied across five databases: PubMed, CINAHL, Embase, Scopus, and Web of Science. These databases were selected to provide broad coverage of biomedical and rehabilitation literature most relevant to MT interventions and physiological outcome measures. While changes in CORT and OXT are often examined within psychological and behavioral research, studies directly investigating MT as a clinical intervention are rarely indexed in these databases; therefore psychological databases such as PsycINFO were not included in this review. Keywords and indexing terms related to CORT and OXT responses, MT interventions, and human subjects were used (see Supplementary Appendix 1). Search results were managed using EndNote 21 and Covidence, with duplicates removed, and the study selection was documented using a PRISMA flow diagram (Figure 1). A review protocol was created through Open Science Framework. In line with several current scoping review recommendations, decisions about the search strategy were shaped by feasibility and focused on peer-reviewed studies to map the published MT evidence base, rather than to comprehensively attempt to capture all possible sources (i.e., grey literature).

### 2.3 Inclusion criteria

Peer-reviewed studies were included if they were published in English, involved non-cadaveric human subjects, reported original data, implemented MT or simulated MT interventions (e.g., massage, osteopathic manipulative treatment, manipulation), and reported at least one physiological outcome related to CORT and/or OXT. Case reports and small case series were also included if they met these criteria. These criteria were applied to facilitate consistent extraction of study characteristics within the scope of this review.

### 2.4 Exclusion criteria

Studies were excluded if they were categorized at practice guidelines, unpublished manuscripts, dissertations, reviews, expert commentaries, books or book chapters, government reports, or conference proceedings. Additional exclusions included studies relating to pregnancy, lactation, newborn/infants, as well as those involving combination treatments where MT was delivered in combination with another therapeutic intervention. However, if studies with multiple interventions had a group receiving only MT, then the results pertaining to this particular subgroup were included in the review.

### 2.5 Screening procedure

A two-phase screening process was conducted. Phase I involved title and abstract screening to remove irrelevant studies. Phase II involved full-text screening to assess eligibility. Both phases were performed independently by two reviewers (ATC, MSA), with disagreements resolved by a third reviewer (WRR).

### 2.6 Data extraction

The following data were extracted from each eligible study: author(s), year of publication, study purpose, keywords, language, population, type of MT or simulated MT, intervention parameters, and primary physiological outcomes. Data extraction parameters were defined *a priori* by two authors (ATC, MSA). Extraction was conducted by one author (ATC) and verified by a second (WRR) to minimize error.

### 2.7 Data synthesis

Findings were summarized descriptively according to several domains. Numerical mapping was used to summarize the total number of included studies, examine trends by year of publication, and describe the distribution of evidence according to hormone measured and choice of biological sampling, MT intervention type, and population characteristics. Intervention-level summaries captured reported delivery parameters and associated physiological responses within each MT approach. Consistent with scoping review methodology, formal appraisal of study quality or risk of bias was not conducted. The scoping review optional consultation stage to generate practice recommendations or stakeholder consensus as described by Arksey and O'Malley and/or others was not undertaken for this review.

## 3 Results

### 3.1 Cortisol—thrust manipulation

Manual therapy studies demonstrating non-significant and significant changes in cortisol post-intervention are shown in Tables 1, 2. The following text summarizes key studies and significant findings. Valera-Calero et al., compared the effects of cervical manipulation (*n* = 28), cervical mobilization (*n* = 28), and cervical sham treatment (*n* = 27) on saliva cortisol (sCORT) levels in 83 adults with chronic mechanical neck pain. sCORT was assessed at baseline, immediately after treatment, and at one week follow-up. A significant and comparable increase in sCORT was found immediately after the intervention in both the cervical manipulation and cervical mobilization group. In contrast, a significant decrease was yielded in the sham manipulation group (18). Other studies also report short-term increases in CORT release following spinal manipulation (17).

**Table 1:** Cortisol non-significant (N.S.) changes.

Author/s Year	Sample size	Intervention	Duration	Symptoms	Biological Measures	Outcome
Adib-Hajbaghery et al. (2013) (44)	60 adults	Whole body massage	60 min—1 session	Patients in coronary care unit	Serum	CORT N.S.
Arroyo-Morales et al. (2009 (45)	60 adults	Massage	40 min—single session	Healthy	Saliva	CORT N.S.
Bennett et al. (2016) (46)	36 students	Massage—Thai	90 min—1 session	Stress	Saliva	CORT ↓ but so did the control
Bost and Wallis (2006) (47)	60	Massage—Swedish	15 min—1/wk—5 wks	Healthy	Urine	CORT N.S.
Christian et al. (1988) (48)	40 males	Spinal manipulative therapy	1 session	Acute spinal pain	Plasma	CORT N.S.
de Oliveira et al. (2018) (49)	24 adult females	Massage—Swedish	40 min—2/wk—3 months	Fibromyalgia	Saliva	CORT N.S.
Donoyama et al. (2010) (50)	15 females	Massage—Anma	40 min—2/wk—4 wks	Healthy	Saliva	CORT N.S.
Donoyama and Shibasaki (2010) (51)	8 females	Massage—Anma	40 min—1 session	Muscle stiffness	Saliva	CORT ↓ but so did control
Donoyama and Norio Toyomi. (2011) (52)	10 females	Massage—Japanese	Eight 40 min/2 wks	Cancer survivors vs. Healthy	Saliva	CORT N.S.
Henderson et al. (2010) (53)	17 adults	Rib raising	5 min	Healthy	Saliva	CORT N.S.
Hernandez-Reif et al. (2001) (54)	24	Massage	30 min—2/wk—5 wks	Low back pain	Urine	CORT N.S.
Hernandez-Reif et al. (2002) (55)	16 adults	Massage	30 min—2/wk—5 wks	Parkinson’s disease	Urine	CORT N.S.
Hernandez-Reif et al. (2004) (56)	34 women	Massage	30 min—3/wk—5 wks	Stage 1/2 breast cancer	Urine	CORT N.S.
Khilnani et al. (2003) (57)	30 adolescents	Massage	20 min—2/wk -1 month	ADHD	Saliva	CORT N.S.
Kim et al. (2001) (58)	59 adults	Hand massage	20 min	Cataract surgery	Blood (not specified)	CORT N.S.
Kovanur-Sampath et al. (2017) (34)	24 males	Thoracic manipulation	1 session	Healthy	Saliva	CORT N.S.
Kucuk Alemdar et al. (2023) (59)	99 adolescents	Hand massage	20 min	Pediatric ICU	Serum	CORT N.S.
Lindgren et al. (2013) (60)	20	Touch massage	60 min—1 session	Anxiety	Serum	CORT N.S.
Lohman et al. (2019) (16)	28 females	Cervical spine manipulation	1 session	Non-specific mechanical neck pain	Serum	CORT N.S., OXT ↑
Lovas et al. (2002) (61)	2 female adults	Massage—Swedish	2x—1 hour—1/wk, 4 wks	Healthy	Serum	CORT N.S.
McRee et al. (2003) (62)	42 adults	Massage—Swedish	1 session—30 min	Surgery patients	Serum	CORT N.S.
Noto et al. (2010) (63)	25 young females	Back massage	10 min	Healthy	Saliva	CORT N.S.
Nuno et al. (2019) (64)	2 adults	Osteopathic manipulative treatments	30 min—3 session/7 wks	Healthy	Urine	N/A
Post-White et al. (2009) (65)	23 children	Massage	∼30 min—4/wk	Cancer—chemotherapy	Saliva	CORT N.S.
Rapaport et al. (2010) (66)	53 adults	Massage—Swedish	45 min—1 session	Healthy	Saliva + Serum	CORT N.S.
Rapaport et al. (2012) (6)	45 young adults	Massage—Swedish	45 min	Healthy	Saliva + Plasma	CORT N.S.
Schaub et al. (2018) (67)	40	Hand massage	16-20 min—7 sessions over 3 wks	Agitated patients with dementia	Saliva	CORT N.S.
Silva et al. (2020) (68)	40	Suboccipital fascial release	5 min—1 session	Healthy	Saliva	CORT ↓ but so did the control
Taylor et al. (2003) (69)	105 females	Massage—Swedish	45 min—3 consecutive evenings	Postsurgical pain, negative affect, and stress	Urine	CORT N.S.
Wändell et al. (2010) (70)	53 adults	Tactile massage	1 hour	Type 2 diabetes	Urine	CORT N.S.
Whelan et l. (2002) (71)	30 males	Cervical spine manipulation	1 session	Healthy	Saliva	CORT N.S.

Tables are organized first by CORT and OXT followed by alphabetically. Up and down arrows represent significant increases and decreases respectively, and N.S. denotes no significance.

**Table 2:** Cortisol significant changes.

Author/s Year	Sample size	Intervention	Duration	Symptoms	Biological Measures	Outcome
Adib-Hajbaghery et al. (2015) (72)	90 males	Massage	60 min—1 session	CVD	Serum	CORT ↓
Andersson, K. (2004) (73)	11 females	Tactile massage	60 min—1/wk—10 wks	Type 2 diabetes	Blood (nots specified)	OXT N.S. but CORT ↓
Boghrabadi et al. (2017) (74)	24 females with type 2 diabetes	Massage	30 min—3/week—1 month	Diabetic	Serum	CORT ↓ in the experimental group after 12 sessions (*p* < 0.05). However, between-group results yield N.S. group.
Aparecida Martins (2023) (75)	41	Massage—Shiatsu		Limb fracture	Saliva	CORT ↓
Ditzen et al. (2007) (76)	67 females	Massage	10 min—1 session	Stressed	Saliva (CORT) + Plasma (OXT)	CORT ↓, OXT N.S.
Farrell et al. (2023) (77)	20 adults	Cervical spine mobilization	12 min (6 min/segment—2 segments)	Healthy	Saliva	CORT ↓
Farrell et al. (2024) (78)	19 adult males	Cervical spine mobilization	12 min (6 min/segment—2 segments)	Persistent-concussion symptoms	Saliva	CORT ↓
Field et al. (1992) (79)	72 children	Massage	30 min—1/ day—5 days	Depressed + adjustment disorder	Saliva + Urine	sCORT ↓, uCORT ↓, but only in the depressed group
Field et al. (1996) (80)	32 females	Massage	30 min—2/wk—5 wks	Depressed	Saliva + Urine	CORT ↓
Field et al. (1997) (81)	20 children	Massage—stroking	15 min/day—1 month	Juvenile rheumatoid arthritis	Saliva	CORT ↓
Field et al. (1997) (82)	20 females	Massage	30 min—2/week—1 month	Sexual abuse	Saliva	CORT ↓ but only on the last day
Field et al. (1998) (83)	32 children	Massage	20 min/day—30 days	Asthma	Saliva	CORT ↓ after massage in younger children (4-8 years old) but not older children (9-14 years old) compared to control
Field et al. (1998) (84)	28 adults	Massage	20 min—daily for 1 wk	Burn injuries	Saliva	CORT ↓
Field et al. (1998) (85)	24 female adolescents	Massage	30 min—2/wk—5 wks	bulimic inpatients	Saliva + Urine	CORT ↓ in massage group but only in urine
Field et al. (1998) (86)	10 geriatric	Massage—Swedish	30 min—3/wk—3 wks	Healthy	Saliva + Urine	CORT ↓ but only long term (urine)
Garner et al. (2008) (87)	32 young adults	Massage	20 min—daily—7 wks	Psychiatric young adults	Saliva	CORT ↓
Hart et al. (2001) (88)	19 females	Massage	30 min—2/wk—5 wks	Anorexia	Saliva + Urine	CORT ↓
Hernandez-Reif et al. (2000) (89)	30 adults	Massage	30 min- 2/wk—5 wks	High BP	Saliva + Urine	CORT ↓
Hodgson and Lafferty (2012) (90)	18 geriatric	Massage—Swedish	20 min—1 session	Cancer	Saliva	CORT ↓
Ironson et al. (1996) (91)	29 gay males	Massage	45 min—5/wk—4 wks	HIV+, HIV-	Saliva	CORT ↓
Lawler and Cameron (2006) (92)	47	Massage	45 min—1/wk—6 wks	Migraine	Saliva	CORT ↓
Leivadi et al. (1999) (93)	30 females	Massage	30 min—2/wk—5 wks	Healthy	Saliva	CORT ↓
Maratos et al. (2017) (94)	139 females	Hand massage	7 min—1 session	Healthy	Saliva	CORT ↓
Padayachy et al. (2010) (17)	30 adult males	Spinal manipulative therapy	Unspecified	Low back pain	Serum	CORT ↓
Pala et al. (2024) (95)	39 youth	Osteopathic sympathetic harmonization	1 session	Major depressive disorder	Saliva	CORT ↑ at 20 min after intervention
Pinar and Afsar (2016) (96)	44	Massage	15 min—7/wk—1 wk	Cancer	Plasma	CORT ↓
Plaza-Manzano et al. (2014) (97)	30 adults	Cervical vs thoracic manipulation	1 session	Healthy	Serum	OXT ↑ CORT increased in cervical group but ↓ at 2-hour post intervention
Stringer et al. (2008) (98)	39 patients	Massage	20 min	Chemotherapy	Serum	CORT ↓
Sunshine et al. (1996) (99)	30 adults	Massage	30 min—2/wk—5 wks	Fibromyalgia	Saliva	CORT ↓
Törnhage et al. (2013) (100)	45 adults	Tactile massage	52 min (Avg) — 10 sessions over 8 wks	Parkinson's disease	Saliva	CORT ↓ within both groups but N.S. between group
Tuchin (1998) (101)	9	Chiropractic manipulation	4 treatments over 2 wks	Healthy	Saliva	CORT ↓ but after the exclusion of 1 outlier participant
Valencia et al. (2025) (102)	10 medical students	Osteopathic manipulative treatments	15 min—1/wk—6 wks	Stressed	Saliva	CORT ↓
Valera-Calero et al. (2019) (18)	83 adults	Cervical manipulation vs cervical mobilization	1 session manipulation/3 min mobilization	Healthy	Saliva	CORT ↑
Wojcik et al. (2022) (103)	57 males	Craniosacral therapy	20 min—1/wk—5 wks	Healthy	Serum	CORT ↓

In contrast to spinal manipulation studies reporting significant changes in CORT post-intervention, several spinal manipulation studies failed to demonstrate CORT changes (16, 48, 71, 104). Lohman et al. investigated serum CORT changes immediately following cervical manipulation on 28 females with non-specific neck pain and reported no significant changes compared to baseline. Similar results of a lack of serum CORT changes were reported following thoracic manipulation of 40 males (20 symptomatic) at 5- or 30-minutes post-manipulation (48). In addition, no sCORT changes were reported in 24 men receiving thoracic manipulation at 5 min, 30 min, and 6 h post-intervention when compared to baseline. Whelan et al. (71) reported significant decreases in sCORT after a single manipulation; however, decreases were present in all three study groups (control, sham-manipulation, manipulation) and were attributed to diurnal rhythms as opposed to the manual therapy intervention itself.

Studies examining CORT responses following thrust spinal manipulation demonstrated variable outcomes across study designs and populations. Reported differences were observed with respect to participant symptomatology, intervention protocols, and the timing and medium of CORT sampling. Collectively, studies of thrust manipulation more frequently report short-term increases in CORT, although findings remain inconsistent across studies that differ in sampling time points, methodologies, and participant populations.

### 3.2 Cortisol—non-thrust MT

Among non-thrust MT studies in adults, participants that received tactile massages (60 min, 1/week, 10 weeks) and had blood collected before massage treatment, one week after, and 12 weeks after found CORT levels significantly decreased only between pre-intervention and 1 week after the intervention (73). Adib-Hajbaghery et al. compared the differences between massage applied by a nurse specialist versus a patient's relative on serum CORT levels. Participants were all male hospital patients and were split into three groups: massage by nurse, massage by patient's relative, and control (routine care). Each massage group received a single session 60 min massage. Serum CORT levels significantly decreased only when the massage was administered by a nurse specialist (72). Leivadi et al. examined the effects of 30 min massages (2x/week for 5 weeks) on sCORT in 30 female dancers with sCORT significantly decreasing between pre and post intervention on both first and last day with the control group not reporting any significant changes (93). Other massage studies reporting decreases in sCORT include studies: in individuals with migraine (92), in psychiatric inpatient adults (87), cancer patients (90), healthy adults (6), females (94), in gay men (91), couples (76), companions of individuals in a coronary care unit (44), individuals undergoing chemotherapy (98), caregivers of cancer patients (96), juveniles with mild-to-moderate rheumatoid arthritis (81), in retired volunteers delivering Swedish massage to infants (86), in bulimic, inpatient females (85), in burn patients (84), in suboccipital release (68), craniosacral therapy (103), in medical students (102), in individuals with post-concussion symptoms (78), in sexually abused women (82), in diabetic women (105), in children psychiatric patients (79), in children with asthma (83), and in individuals post-operatively [(75), (Table 2)]. It is noteworthy that in healthy males receiving either upper or lower cervical mobilization, that a significant reduction of sCORT was reported 30 min only after the lower cervical mobilization for both within and between groups, suggesting anatomical location of mobilization may perhaps influence sCORT response (77). In a study of similar design by the same investigators in people with post-concussion symptoms receiving either upper or lower cervical mobilization, only lower cervical mobilization resulted in a significant within group reduction (5 min before compared to 30 min after intervention) in sCORT, however no significant between group differences were found at post 5 or 30 min (78).

It is also noteworthy that several studies report significant decreases in sCORT in both the intervention and control groups including studies in individuals with Parkinson's disease (100), and in 50-year-old women who received Anma massage from different proficiency level practitioners (50). Significant decreases in sCORT in the intervention and relaxation control groups were similarly reported following a single Thai massage session in 36 self-perceived academically stressed students (46). Participants were randomized into the intervention group (1 session, 5 min, *n* = 15) or control (laid down). sCORT was analyzed with the first five minutes preceding the intervention and during the first 5 min immediately after the intervention and yielded significant decreases in both groups (68).

Similar to studies involving spinal manipulation, many massage studies also reported a lack of significant changes in CORT post-intervention. Studies reporting a lack of CORT changes following massage included: adolescents in intensive care (59), children undergoing chemotherapy (65), adult cancer survivors (52), healthy adults (57, 61, 63, 66), adults with chronic muscle stiffness following Japanese massage (50), individuals post-exercise (45), individuals with fibromyalgia (49, 99), dementia (67), hypertension (89), women with anorexia nervosa (88), individuals post-op (60, 62, 69), and one study reported CORT increases in the control group but not in the hand massage group (58).

Among studies investigating changes in urinary cortisol (uCORT) levels following massage, Field et al. compared the effects of massage and relaxation on uCORT and sCORT levels in depressed adolescent mothers. Participants either received a 30-min massage intervention (2x/week, 5weeks) or participated in relaxation techniques (15-min yoga and 15-min breathing exercises, 2x/week, 5 weeks). Saliva was collected 30 min before the session, immediately after, and 30 min after the session on the first and last day of intervention. Urine was collected immediately after the intervention on the first and last day. uCORT yielded significant decreases on both first and last day and sCORT levels also significantly decreased between the first and last day. The relaxation control group did not yield any significant changes (80). Hernandez-Reif et al. (55) examined the effects of massage therapy on uCORT and sCORT in 30 adults with hypertension. sCORT was assessed before and 20 min after the intervention on the first and last day, and uCORT was assessed before the intervention on the first and last day. Compared to the control group, only the massage group showed significant decreases in both uCORT and sCORT (89). Bost et al. (47) investigated the effectiveness of Swedish massage in reducing physiological and psychological stress indicators in 60 nurses at an acute care hospital. Nurses received back massages (15 min, 1x/week, 5/weeks) with urine samples collected in the morning during the 1st, 3rd, and 5th weeks. No significant changes in uCORT levels occurred between week one and five in the massage group compared to control (47). Several other massage studies also reported no changes in uCORT levels including in a diabetic population (70), individuals with low back pain (54), Parkinson's disease (55), women with anorexia nervosa (88), and early-stage breast cancer (56).

Studies examining CORT responses following non-thrust MT reported heterogeneous outcomes across intervention types and study populations. In contrast to thrust manipulation, non-thrust MT approaches (particularly massage-based interventions), more frequently reported decreases in CORT. However, these effects were observed across diverse populations and study protocols, limiting comparability.

### 3.3 Oxytocin

Compared to CORT, fewer studies have investigated the effects of MT on OXT levels (Tables 3, 4). Morhenn et al. examined the effects of a single Swedish massage on OXT levels in 95 healthy adults. Participants were split into the massage group (*n* = 65, 1 session, 15 min) or control (*n* = 30, rest). Serum OXT was assessed immediately before (*n* = 95), with a subset (*n* = 24) having it assessed immediately after the massage session. The remaining participants in the massage group (*n* = 41) had their serum assessed after completing an economic task. This study design was implemented to ensure OXT levels were not being influenced from monetary gain. The massage group yielded a significant increase in serum OXT levels (33). Tsuji et al., examined the effects of massage on salivary OXT levels in mothers and children after massage. Children were massaged by their mothers for 20 min/day, every day, for 3 months. After the massage phase, they then had a 4-month phase of no massage for 4 months. Salivary OXT assessments were made before and 20 min after the intervention every 3 weeks during the massage phase and every 4 weeks during the no-massage phase. During the massage phase, salivary OXT levels were significantly higher (but only when averaging basal OXT levels throughout the entire phase), compared to the no massage phase. Significant increases were not found after a single intervention, suggesting an accumulation effect with OXT (111). In another interesting study comparing hand-delivered versus machine-delivered 10-min foot massages, both resulted in significantly increased plasma OXT levels immediately post-intervention, but more potent OXT changes (51.8% vs 18.2%) were reported after hand massage, which was deemed more pleasurable and rewarding (110). Hand massage also demonstrated greater cortical activation of regions processing reward aspects of social touch than machine-delivered massage as accessed using functional near infrared spectroscopy (110).

**Table 3:** Oxytocin non-significant changes.

Author/s Year	Sample size	Intervention	Duration	Symptoms	Biological Measures	Outcome
Andersson et al. (2004) (73)	11 females	Tactile massage	60 min—1/wk—10 wks	Type 2 diabetes	Serum	OXT N.S. but CORT ↓ between week 1 + week 2
Bello et al. (2008) (106)	14 males	Massage	20 min	Healthy	Serum	OXT N.S.
Ditzen et al. (2007) (76)	67 females	Massage	10 min—1 session	Stressed	Saliva (CORT) + Plasma (OXT)	OXT N.S. but CORT↓
Henricson et al. (2008) (107)	44 adults	Tactile touch	1 hour—1/day—5 days	ICU patients	Serum	OXT N.S. changes
Rapaport et al. (2010) (66)	53 adults	Massage—Swedish	45 min—1 session	Healthy	Saliva + Serum	Both N.S.
Rapaport et al. (2012) (6)	45 young adults	Massage—Swedish	45 min	Healthy	Saliva + Plasma	Both N.S.
Turner et al. (1999) (108)	24 females	Massage—Swedish	15 min—1 session	Healthy	Plasma	OXT N.S.
Wikström et al. (2003) (109)	22 adults	Massage—Swedish	30 min	Healthy	Plasma	OXT N.S.

**Table 4:** Oxytocin significant changes.

Author/s Year	Sample size	Intervention	Duration	Symptoms	Biological Measures	Outcome
Li et al. (2019) (110)	40 adult males	Foot massage	10 min	Healthy	Plasma	OXT ↑ in both hand-administered (*p* < 0.0001) and machine (*p* = 0.02), but hand was more potent
Lohman et al. (2019) (16)	28 females	Cervical spine manipulation	1 session	Neck pain	Serum	OXT ↑, CORT N.S.
Morhenn et al. (2012) (33)	95 adults	Massage	15 min—1 session	Healthy	Serum	OXT ↑
Plaza-Manzano et al. (2014) (97)	30 adults	Cervical vs thoracic manipulation	1 session	Healthy	Serum	OXT ↑, CORT increased in cervical group but ↓ at 2-h post intervention
Tsuji et al. (2015**)** (111)	7 children	Massage—touch therapy	20 min/day—3 months	Autistic	Saliva	OXT ↑

Wikstrom et al., examined the effects of Swedish massage on plasma OXT in 22 healthy adults. Participants were given a single session of a Swedish massage (30 min) and had serum OXT assessed immediately before, after 60 min after the intervention. Serum OXT yielded no significant differences, although they did note that OXT were varied widely between men and women at baseline (109). In a randomized controlled trial, Henricson et al., examined the effects of five-day tactile touch intervention on serum OXT in 44 adults in the intensive care unit. Participants were split into either the tactile touch group (1 h/day, 5 days) or control (standard care). Serum oxytocin was collected before (noon) and after the intervention and standard treatment (1pm). Serum OXT levels did not significantly change in the touch group; in contrast, the standard care group saw a significant increase (107). Other studies also reported a lack of OXT changes following massage (76).

### 3.4 Studies measuring both cortisol and oxytocin

A small subset of studies assessed both CORT and OXT responses following MT interventions. Plaza-Manzano et al. examined the effects of cervical and thoracic spinal manipulation in asymptomatic adults and reported significant increases in OXT immediately following both interventions, with sustained elevations at two hours in the cervical manipulation. In contrast, CORT levels demonstrated a transient increase immediately post-manipulation followed by a significant decrease at two hours in the cervical manipulation group (97). Rapaport et al. compared the effects of a single session of Swedish massage to a light touch control in healthy adults and assessed both plasma and salivary CORT along with plasma OXT at multiple time points post-intervention (6, 66). No significant changes were observed in either CORT or OXT following massage compared to control (66). Lohman et al. reported increases in OXT following cervical manipulation, but no changes in CORT (16). Two studies reported a significant decrease in CORT while yielding no significant changes in OXT (73, 76).

## 4 Discussion

Consistent with the Arksey and O'Malley framework (39) and subsequent refinements described by others (35, 36, 41), this scoping review mapped the existing literature examining CORT and OXT responses following three distinct MT treatment approaches (spinal manipulation, joint mobilization, massage). A key strength of this review is its inclusion of multiple MT modalities rather than focusing on a single MT intervention type. Collectively, this review highlights the substantial variability in reported MT-related hormone/neuropeptide responses, underscoring the future importance of MT standardization, addressing knowledge gaps, and resolving methodological challenges noted within the existing evidence base.

### 4.1 Key patterns and evidence gaps in neuroendocrine responses to MT

Despite MT having been long proposed to influence CORT and OXT through inherent physical touch, resultant muscle relaxation and/or stress reduction; when the evidence is examined collectively, findings across MT studies remain mixed and highly variable. Among investigations assessing CORT, approximately half reported significant changes, while the remainder reported either no significant effects or similar changes occurring in both the intervention and control groups. Notable, studies involving thrust manipulation more often reported short-term increases in CORT, whereas non-thrust MT approaches more frequently demonstrated decreases in CORT across diverse study populations. These differences in the direction of CORT responses by different MT techniques suggest that distinct physiological processes may be engaged which warrants further investigation.

**Figure 2:**
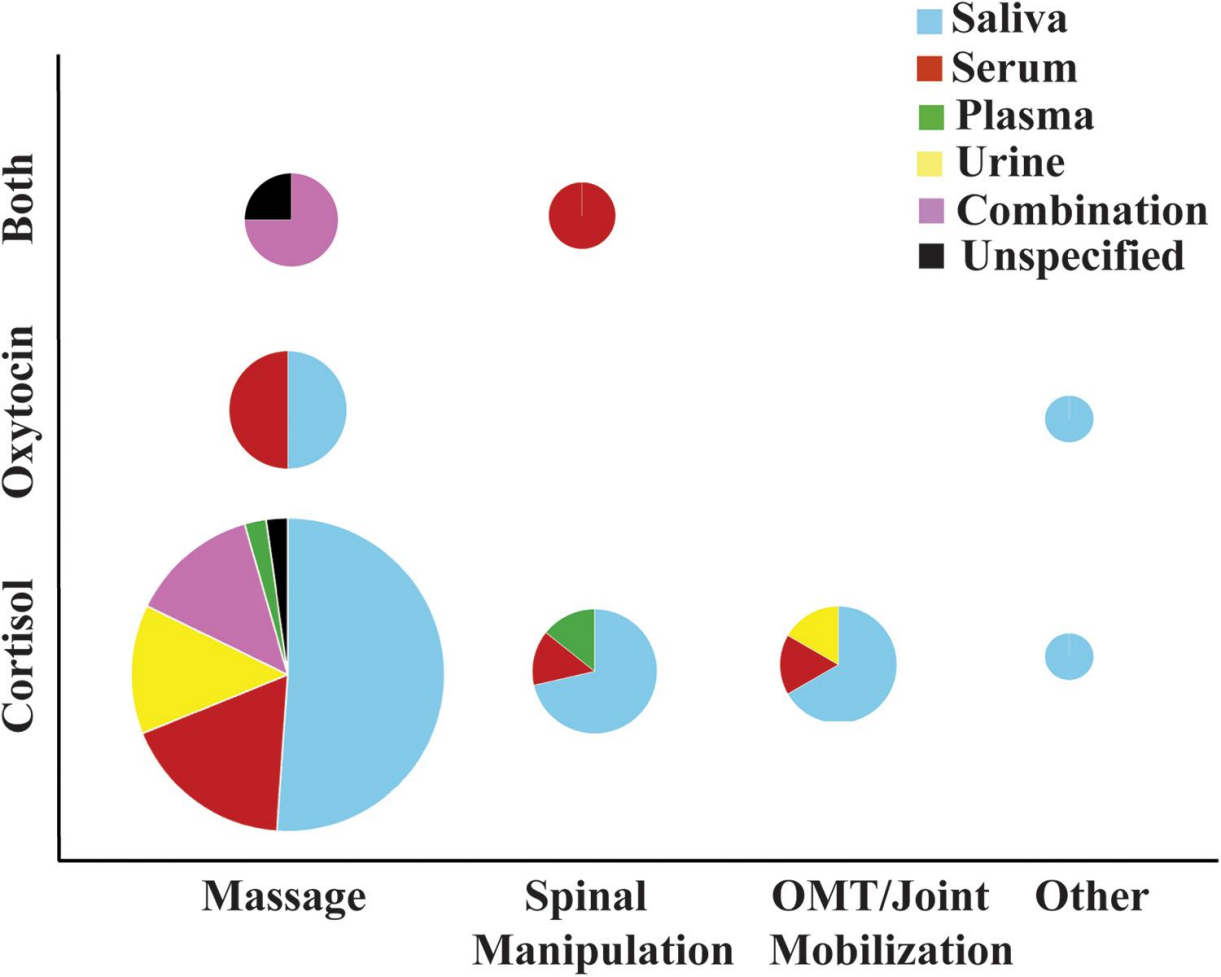
Illustration of the type and proportion of manual therapy intervention studies along with the biological sampling methodology of the specific neuroendocrine hormones.

Many of the CORT studies focused on Swedish massage; a technique primarily aimed at promoting relaxation and relieving tension. This modality typically involves long, gliding strokes, kneading, and friction to warm the muscles, enhance circulation, and reduce physical stress (47, 112). If a study broadly referred to the intervention as “massage” or “standard massage” without specifying the technique, it was assumed to be Swedish. Totaling 35 of the studies, 20 of them yielded significant changes in CORT levels between pre- and post- intervention. Notable variation exists across these studies, particularly in terms of massage duration, which ranged from 10 to 90 min, and in treatment frequency, which spanned from a single session to multiple sessions per week over a 12-week period (46, 63, 113). Forty-seven percent of the CORT massage studies performed massage 16-30 min, while 33% performed massage 31-60 min. To our knowledge comparisons of CORT changes based on massage duration has not been performed. While there are far fewer OXT studies (Figure 2), they are more evenly distributed across massage durations. Figure 2 provides a visual representation of the number of CORT and OXT studies performed, and the type of biological sampling involved. The overall lack of OXT MT-related studies is clearly apparent as is the dominance of saliva sampling of neuroendocrine changes (Figure 2). Additionally, the health status and age of the participants varied considerably among CORT and OXT studies, with studies including healthy individuals as well as those with psychiatric conditions, chronic pain, cancer, and other clinical populations. However, neither treatment frequency, age, nor participant health condition appeared to consistently influence whether significant changes were reported for either CORT or OXT levels.

The second most common intervention among the CORT studies was spinal manipulation. Within this MT subset, four studies reported either no significant changes or non-specific results. These study findings also remain inconclusive, and the type of manipulation (i.e., cervical versus thoracic) did not appear to likely influence the likelihood of yielding significant changes. Notably, Plaza-Manzano et al. (97) and Lohman et al. (16) are the only studies to date that examined the effects on both CORT and OXT levels. Both studies reported an increase in OXT but differing results for CORT.

In contrast to CORT studies, substantially fewer studies examined OXT responses following MT with 65 assessing CORT outcomes, 13 assessing OXT, and 6 measuring both hormones. Approximately two-thirds of OXT studies were conducted in healthy participants, while the remainder included symptomatic populations, with no consistent pattern of OXT response observed by participant health status. Similar to CORT, a near equal division of MT findings measuring OXT changes was also found with 5 studies reporting significant changes, while 8 studies reported a lack of significant OXT changes with MT. Both CORT and OXT studies demonstrated large variability in reported findings. This is most likely the result of the lack of standardization regarding the MT application, differences in MT applied forces and MT treatment durations, small sample sizes, and differing methodologies used to sample and process CORT and OXT in the same bodily fluids. Much greater consideration needs to be given to conducting CORT and OXT studies in symptomatic populations (acute and chronic musculoskeletal conditions), since a large number of neuroendocrine MT-related studies were performed in healthy populations. However, with that said neither treatment frequency, participant age, nor participant health condition appeared to consistently influence whether a study reported significant changes in CORT levels following an MT intervention. Studies attempting to compare CORT or OXT changes between distinct MT techniques were practically non-existent. Similarly severe lack of longitudinal CORT and OXT MT-related studies creates a large gap in knowledge, however longitudinal MT studies are extremely difficult to control due to complex neuroendocrine interactions, differences in neuroendocrine production, storage, release, and half-life of these hormones/neuropeptides.

Similar to the CORT studies, most OXT studies involved massage, and findings were mixed. Approximately one-third of the studies reported significant changes in OXT levels, while the remaining studies either found no significant changes or observed similar changes in both the intervention and control groups. The five studies that did not report significant effects involved massage and/or touch therapy; however, only studies that incorporated spinal manipulation yielded significant increases in OXT levels. Details such as treatment frequency, participant age, and health condition did not appear to consistently influence whether a study reported significant changes in OXT levels.

**Figure 3:**
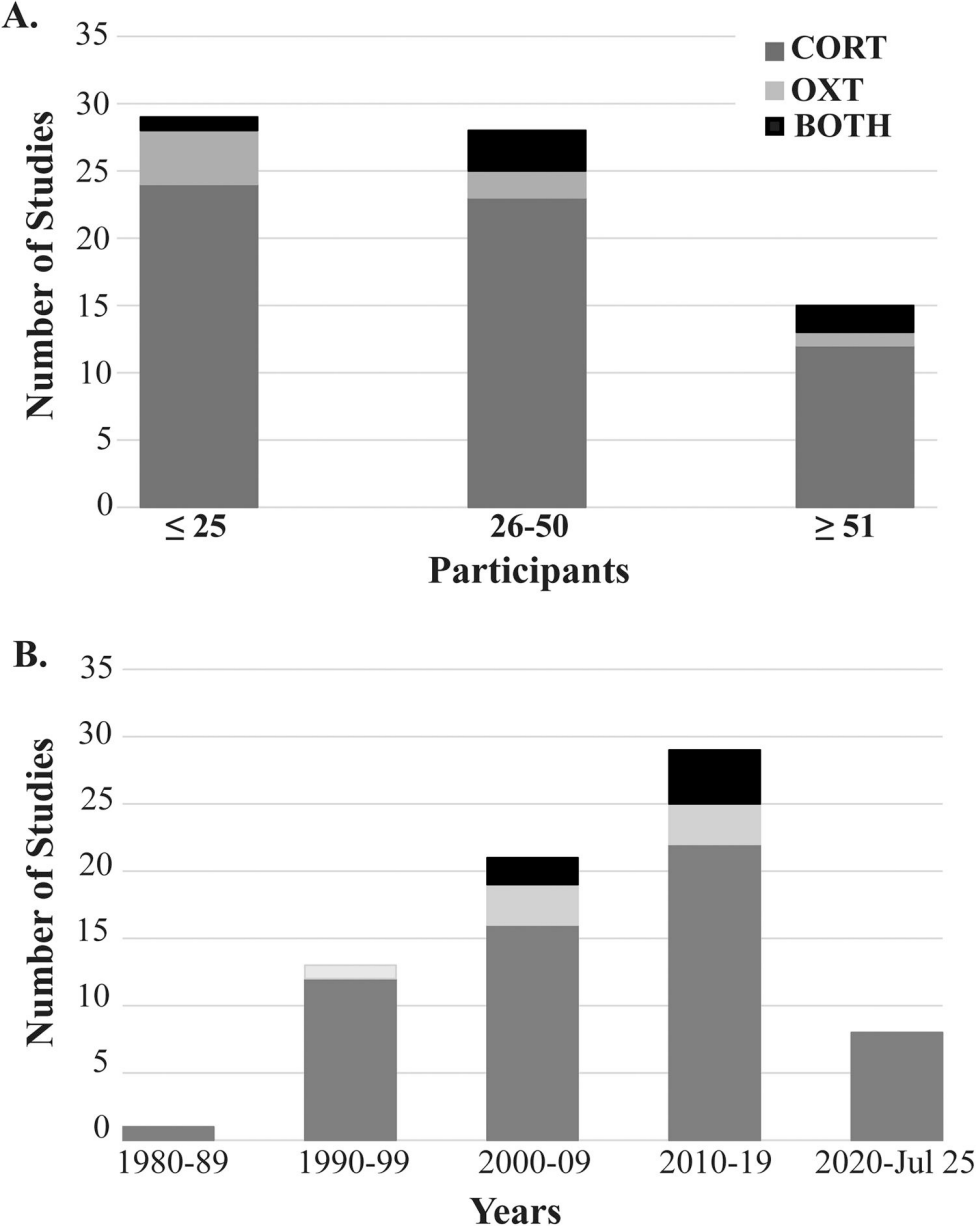
Summaries of the included studies by **(A)** sample size and **(B)** chronological decade with the corresponding number of studies.

To address gaps in knowledge pertaining to CORT and OXT-related MT research, future research should prioritize the development of standardized protocols for MT interventions, including clearly defined MT techniques, treatment durations, and treatment frequencies, to improve consistency across studies. Efforts should also be made to increase sample size and include more diverse populations, as most existing research focuses on small, homogenous samples. As shown in Figure 3A, a majority of CORT and OXT studies were conducted with relatively small sample sizes (<50) which limits statistical power and reduces the likelihood of detecting significant effects. Figure 3B demonstrates a growing interest in hormonal-related manual therapy studies over the last four decades. Ultimately, increasing sample size, advancing methodological rigor and sample collection standardization will be key to identifying potential MT-related neuroendocrine mechanisms and clinical relevance of MT in modulating stress- and pain- related physiology.

### 4.2 Methodological boundaries and sources of heterogeneity

Several important gaps in the literature limit the strength and generalizability of the current MT evidence on CORT/OXT levels. Most MT studies relied on single-session interventions with immediate or short-term follow-up, limiting assessment of delayed or sustained hormonal responses. Variability in the timing and duration of MT administration and biological sample collection further contributed to heterogeneity across studies. While some investigators reported time-of-day and sampling schedules to account for known diurnal variations in CORT, others did not, reducing comparability. Across studies, post-intervention sample collections were variable with 20 min after MT being the most common. Few studies incorporated repeated or longitudinal sampling (6, 67, 73). In addition, one must also remain mindful that OXT is stored in secretory vesicles both within hypothalamic neurons and at axon terminals in the neurohypophysis, allowing for rapid release from pre-existing pools in response to stimulation. While axonal transport contributes to replenishment of these stores, the timing of peripheral oxytocin release following mechanical or sensory stimulation is also variable (31, 114, 115). Interpretation of OXT findings is further complicated by its role as a centrally acting neuropeptide and neurotransmitter, such that peripheral measures may not consistently reflect central activity, as well as by bidirectional interactions between OXT and the HPA-axis (116–118). Support for this concept comes from basic studies, where gentle touch or massage-like stimulation has been shown to activate central OXT signaling and attenuate HPA-axis activity, with effects that depend on the timing and nature of the mechanical stimulation (119–122).

### 4.3 Limitations

Limitations of this review include the exclusion of non-English studies and those examining OXT/CORT in the context of pregnancy, labor, lactation and infancy. The latter decision was made because OXT plays a distinct physiological role in reproductive processes, and including such studies could have only confounded the interpretation of findings related to MT interventions (123). In addition, the inclusion of only peer-reviewed clinical literature may have excluded certain grey literature, which could potentially further inform the hormonal/neuropeptide scope of MT research. Future reviews may also wish to increase the number of databases searched such as PsycINFO to ensure capture of psychological and behaviorally-related studies. The amount of CORT and OXT-related knowledge and insight to be gained from animal studies is large and is deserving of its own review.

## 5 Conclusions

This scoping review mapped the existing literature examining CORT and OXT responses following MT to characterize the scope and nature of the available evidence. While CORT has been examined across a range of MT approaches with mixed results, the evidence base for OXT remains severely limited. Both CORT and OXT MT-related studies demonstrated substantial variability in intervention type, study population, and sample collection/processing, and outcome assessment. This review also helps to identify the extent and shortcomings of MT-related neuroendocrine research and identifies areas where further investigation is needed, particularly with respect to OXT-related studies.

## Data Availability

The original contributions presented in the study are included in the article/Supplementary Material, further inquiries can be directed to the corresponding author.
