# Supplementary material for: Physiological changes of cortisol and oxytocin following manual therapy: a scoping review
Source: Front Rehabil Sci. 2026 Mar 5;7:1719735. doi: 10.3389/fresc.2026.1719735 (PMC13000766; doi:10.3389/fresc.2026.1719735)
Supplement: Supplementary file 1 [file Table1.docx]

Supplemental File 1

**
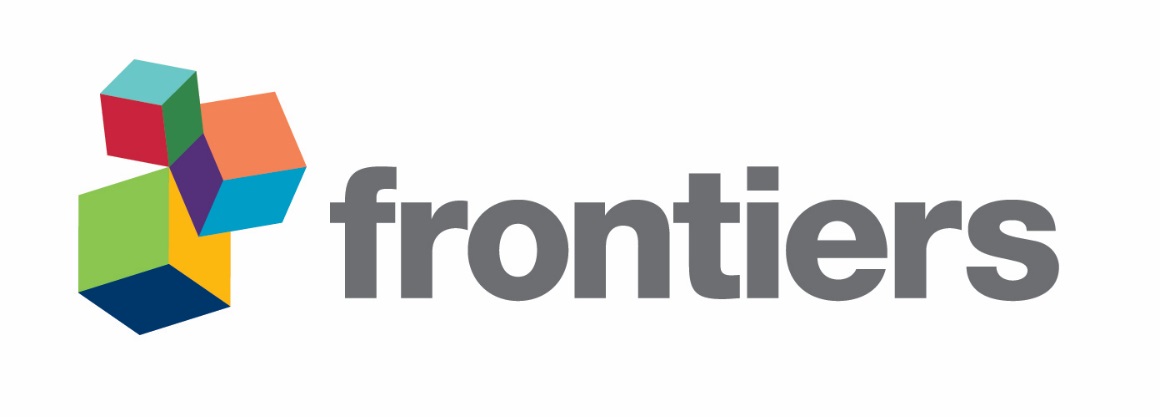
**

Electronic Supplementary Material File S1

Bibliographic Literature searching

| **Database** | **Hits** |
| --- | --- |
| CINAHL Plus with Full Text | 913 |
| Embase | 898 |
| PubMed | 52 |
| Scopus | 351 |
| Web of Science | 160 |
| Total | 2374 |
| - duplicates | 437 |
| Unique studies to screen | 1937 |

Database: CINAHL Plus with Full Text

Host: EBSCOhost

Data Parameters: 1937 to Present

Date Searched: July 14, 2025

Searcher: Rebecca Billings                    

Search Strategy:

| **#** | **Searches** | **Results** |
| --- | --- | --- |
| 1 | (MH "Manipulation, Chiropractic" OR MH "Chiropractic+" OR MH "Manipulation, Orthopedic" OR MH "Manipulation, Osteopathic" OR MH "Research, Chiropractic" OR MH "Chiropractic Practice" OR MH "Massage+" OR MH "Acupressure+" OR MH "Myofascial Release" OR MH "Reflexology" OR MH "Structural-Functional-Movement Integration+" OR MH "Manual Therapy+" OR MH "Instrument-Assisted Soft Tissue Mobilization" OR MH "Physical Therapy+" OR MH "Craniosacral Therapy" OR MH "Applied Kinesiology" OR chiropractic OR HVLA OR "high-velocity low amplitude" OR spinal-manip* OR spinal-mobiliz* OR joint-manip* OR joint-mobiliz* OR fascial-manip* OR fascia-manip* OR massage* OR massaging OR zone-therap* OR "manual lymphatic drainage" or "manual lymph drainage" OR osteopathic-manual* OR osteopathic-manip* OR TI "osteopathic" OR osteopathic-treatment* OR osteopathic-therap* OR cranial-osteopath* OR visceral-osteopath* OR "osteopathic research" OR "osteopathic care" OR osteopathic-intervention* OR reflexolog* OR "applied kinesiology" OR soft-tissue-manip* OR "manual therapy" OR musculoskeletal-manip* OR physical-therap* OR physiotherap* OR bodywork* OR manual-therap* OR manipulative-therap* OR manipulation-therap* OR "myofascial structural integration" OR "myofascial release" OR rolfing OR rolf OR fascia* OR fasciae OR myofascia*) | 266319 |
| 2 | (MH "Oxytocin" OR oxytocin* OR cortisol* OR neurotensin* OR orexin* OR MH "Cytokines+" OR cytokines OR MH "Neuropeptides+" OR neuropeptide*) OR ((MH "Oxytocin" OR oxytocin* OR MH "Hydrocortisone" OR cortisol* OR hydrocortisone*) AND (biochemical* OR endocrine* OR neuroendocrine* OR neurohormone*)) | 155632 |
| 3 | #1 AND #2 | 2951 |
| 4 | #3 NOT (cardiac-massag* OR heart-massag* OR rectal* OR carotid-sinus-massag* OR lactat* OR breast* OR breastfeed* OR newborn* OR caesarean OR pregnan* OR vaginal* OR labor* OR labour* OR postpartum OR hemorrhag* OR haemorrhag* OR high-intensity OR resistance-train*) | 1920 |
| 5 | Filter: Human only | 913 |

Database: Embase

Host: Elsevier

Data Parameters: 1947 to Present

Date Searched: July 14, 2025

Searcher: Rebecca Billings

Search Strategy:

| **#** | **Searches** | **Results** |
| --- | --- | --- |
| 1 | ('acupressure'/de OR 'bodywork'/exp OR 'chiropractic'/de OR 'massage'/exp OR 'musculoskeletal manipulation'/exp OR 'osteopathic medicine'/exp OR 'reflexology'/de OR 'soft tissue therapy'/de OR 'trigger point therapy'/de OR 'physiotherapy'/de OR 'kinesiology'/de OR 'osteopathic':ti OR (chiropractic OR HVLA OR 'high-velocity low amplitude' OR spinal-manip* OR spinal-mobiliz* OR joint-manip* OR joint-mobiliz* OR fascial-manip* OR fascia-manip* OR massage* OR massaging OR zone-therap* OR 'manual lymphatic drainage' or 'manual lymph drainage' OR osteopathic manual* OR osteopathic-manip* OR osteopathic-treatment* OR osteopathic-thera* OR cranial-osteopath* OR visceral-osteopath* OR 'osteopathic research' OR 'osteopathic care' OR osteopathic-intervention* OR reflexolog* OR 'applied kinesiology' OR soft-tissue-manip* OR 'manual therapy' OR musculoskeletal-manip* OR physical-therap* OR physiotherap* OR bodywork* OR manual-therap* OR manipulative-therap* OR manipulation-therap* OR 'myofascial structural integration' OR 'myofascial release' OR rolfing OR rolf OR fascia* OR fasciae OR myofascia*):ab,ti,kw) | 286359 |
| 2 | ('oxytocin'/de OR (oxytocin* OR cortisol* OR neurotensin* OR orexin* OR cytokines OR neuropeptide*):ab,ti,kw) OR (('oxytocin'/de OR oxytocin*:ab,ti,kw OR 'hydrocortisone'/de OR hydrocortisone*:ab,ti,kw OR cortisol*:ab,ti,kw) AND (biochemical* OR endocrine* OR neuroendocrine* OR neurohormone*):ti,ab,kw) | 680815 |
| 3 | #1 AND #2 | 2009 |
| 4 | #3 NOT (cardiac-massag* OR heart-massag* OR rectal* OR carotid-sinus-massag* OR lactat* OR breast* OR breastfeed* OR newborn* OR caesarean OR pregnan* OR vaginal* OR labor* OR labour* OR postpartum OR hemorrhag* OR haemorrhag* OR high-intensity OR resistance-train*) | 1055 |
| 5 | Filter: Humans only | 898 |

Database: PubMed

Host: National Library of Medicine (<http://www.ncbi.nlm.nih.gov/pubmed>)

Data Parameters: 1946 to Present

Date Searched: July 14, 2025

Searcher: Rebecca Billings                      

Search Strategy:

| **#** | **Searches** | **Results** |
| --- | --- | --- |
| 1 | ("Manipulation, Chiropractic"[MeSH Terms] OR "Chiropractic"[MeSH Terms] OR chiropractic[tw] OR "HVLA"[tw] OR "high-velocity low amplitude"[tw] OR "spinal manip*"[tw] OR "spinal mobiliz*"[tw] OR "joint manip*"[tw] OR "joint mobiliz*"[tw] OR "fascial manip*"[tw] OR "fascia manip*"[tw] OR "Massage"[MeSH Terms] OR massage*[tw] OR massaging[tw] OR zone-therap*[tw] OR "Manual Lymphatic Drainage"[tw] OR "Manual Lymph Drainage"[tw] OR "Manipulation, Osteopathic"[Mesh] OR "osteopathic manual*"[tw] OR "osteopathic manip*"[tw] OR "osteopathic"[tw] OR "osteopathic treatment*"[tw] OR "osteopathic thera*"[tw] OR "cranial osteopath*"[tw] OR "visceral osteopath*"[tw] OR "osteopathic research"[tw] OR "osteopathic care"[tw] OR "osteopathic intervention"[tw] OR "Musculoskeletal Manipulations"[Mesh] OR "Kinesiology, Applied"[Mesh] OR reflexolog*[tw] OR "applied kinesiology"[tw] OR "soft tissue manip*"[tw] OR "manual therapy"[tw] OR "musculoskeletal manip*"[tw] OR "physical therap*"[tw] OR "Physical Therapists"[Mesh] OR physiotherap*[tw] OR bodywork*[tw] OR manual-therap*[tw] OR manipulative-therap*[tw] OR manipulation-therap*[tw] OR "myofascial structural integration"[tw] OR "myofascial release"[tw] OR rolfing[tw] OR rolf[tw] OR "Fascia"[Mesh] OR "Myofascial Pain Syndromes"[Mesh] OR fascia*[tw] OR fasciae[tw] OR myofascia*[tw]) | 184585 |
| 2 | ("Oxytocin"[MeSH] OR oxytocin*[tw] OR "Hydrocortisone"[Mesh] OR hydrocortisone*[tw] OR cortisol*[tw] OR neurotensin*[tw] OR orexin*[tw] OR cytokines[tw] OR neuropeptide*[tw]) OR ("Oxytocin"[MeSH] OR oxytocin*[tw] OR cortisol*[tw] OR "Hydrocortisone"[Mesh] OR hydrocortisone*[tw]) AND (biochemical*[tw] OR endocrine*[tw] OR neuroendocrine*[tw] OR neurohormone*[tw]) | 39641 |
| 3 | #1 AND #2 | 89 |
| 4 | #3 NOT (cardiac-massag*[tiab] OR heart-massag*[tiab] OR rectal*[tiab] OR "carotid sinus massag*"[tiab] OR lactat*[tiab] OR breast*[tiab] OR breastfeed*[tiab] OR newborn*[tiab] OR pregnan*[tiab] OR vaginal*[tiab] OR labor*[tiab] OR labour*[tiab] OR postpartum[tiab] OR hemorrhag*[tiab] OR haemorrhag*[tiab]) | 64 |
| 5 | Filter: Humans | 52 |

Database: Scopus

Host: Elsevier

Data Parameters: 1960 to Present

Date Searched: July 14, 2025

Searcher: Rebecca Billings

Search Strategy:

| **#** | **Searches** | **Results** |
| --- | --- | --- |
| 1 | TITLE-ABS(chiropractic OR HVLA OR {high-velocity low amplitude} OR spinal-manip* OR spinal-mobiliz* OR joint-manip* OR joint-mobiliz* OR fascial-manip* OR fascia-manip* OR massage* OR massaging OR zone-therap* OR {manual lymphatic drainage} OR {manual lymph drainage} OR osteopathic-manual* OR osteopathic-manip* OR osteopathic OR osteopathic-treatment* OR osteopathic-thera* OR cranial-osteopath* OR visceral-osteopath* OR {osteopathic research} OR {osteopathic care} OR osteopathic-intervention* OR reflexolog* OR {applied kinesiology} OR soft-tissue-manip* OR musculoskeletal-manip* OR physical-therap* OR physiotherap* OR bodywork* OR manual-therap* OR manipulative-therap* OR manipulation-therap* OR {myofascial structural integration} OR {myofascial release} OR rolfing OR rolf OR fascia* OR fasciae OR myofascia*) | 191646 |
| 2 | TITLE-ABS(oxytocin* OR cortisol OR neurotensin OR orexin OR cytokines OR neuropeptides) OR (neuroendocrine AND oxytocin) OR TITLE-ABS((oxytocin* OR cortisol* OR hydrocortisone*) AND (biochemical* OR endocrine* OR neuroendocrine* OR neurohormone*)) | 722158 |
| 3 | #1 AND #2 | 1226 |
| 4 | #3 AND NOT TITLE-ABS(cardiac-massag* OR heart-massag* OR rectal* OR rectum OR carotid-sinus-massag* OR lactat* OR breast* OR breastfeed* OR newborn* OR pregnan* OR vaginal* OR labor* OR labour* OR postpartum OR hemorrhag* OR haemorrhag*) | 903 |
| 5 | Filter: Human; humans; Document Type: Article; Exclude all animal studies | 351 |

Database: Web of Science

Host: Clarivate

Data Parameters: 1990 to Present

Date Searched: July 14, 2025

Searcher: Rebecca Billings

Search Strategy:

| **#** | **Searches** | **Results** |
| --- | --- | --- |
| 1 | (((TI=(chiropractic OR HVLA OR 'high-velocity low amplitude' OR spinal-manip* OR spinal-mobiliz* OR joint-manip* OR joint-mobiliz* OR fascial-manip* OR fascia-manip* OR massage* OR massaging OR zone-therap* OR 'manual lymphatic drainage' or 'manual lymph drainage' OR osteopathic-manual* OR osteopathic-manip* OR osteopathic-treatment* OR osteopathic-thera*' OR cranial-osteopath* OR visceral-osteopath* OR 'osteopathic research' OR 'osteopathic care' OR osteopathic-intervention* OR reflexolog* OR 'applied kinesiology' OR soft-tissue-manip* OR musculoskeletal-manip* OR physical-therap* OR physiotherap* OR bodywork* OR manual-therap* OR manipulative-therap* OR manipulation-therap* OR 'myofascial structural integration' OR 'myofascial release' OR rolfing OR rolf OR fascia* OR fasciae OR myofasia*)) OR AB=(chiropractic OR HVLA OR 'high-velocity low amplitude' OR spinal-manip* OR spinal-mobiliz* OR joint-manip* OR joint-mobiliz* OR fascial-manip* OR fascia-manip* OR massage* OR massaging OR zone-therap* OR 'manual lymphatic drainage' or 'manual lymph drainage' OR osteopathic-manual* OR osteopathic-manip* OR osteopathic-treatment* OR osteopathic-thera*' OR cranial-osteopath* OR visceral-osteopath* OR 'osteopathic research' OR 'osteopathic care' OR osteopathic-intervention* OR reflexolog* OR 'applied kinesiology' OR soft-tissue-manip* OR musculoskeletal-manip* OR physical-therap* OR physiotherap* OR bodywork* OR manual-therap* OR manipulative-therap* OR manipulation-therap* OR 'myofascial structural integration' OR 'myofascial release' OR rolfing OR rolf OR fascia* OR fasciae OR myofasia*)) OR AK=(chiropractic OR HVLA OR 'high-velocity low amplitude' OR spinal-manip* OR spinal-mobiliz* OR joint-manip* OR joint-mobiliz* OR fascial-manip* OR fascia-manip* OR massage* OR massaging OR zone-therap* OR 'manual lymphatic drainage' or 'manual lymph drainage' OR osteopathic-manual* OR osteopathic-manip* OR osteopathic-treatment* OR osteopathic-thera*' OR cranial-osteopath* OR visceral-osteopath* OR 'osteopathic research' OR 'osteopathic care' OR osteopathic-intervention* OR reflexolog* OR 'applied kinesiology' OR soft-tissue-manip* OR musculoskeletal-manip* OR physical-therap* OR physiotherap* OR bodywork* OR manual-therap* OR manipulative-therap* OR manipulation-therap* OR 'myofascial structural integration' OR 'myofascial release' OR rolfing OR rolf OR fascia* OR fasciae OR myofasia*)) OR KP=(chiropractic OR HVLA OR 'high-velocity low amplitude' OR spinal-manip* OR spinal-mobiliz* OR joint-manip* OR joint-mobiliz* OR fascial-manip* OR fascia-manip* OR massage* OR massaging OR zone-therap* OR 'manual lymphatic drainage' or 'manual lymph drainage' OR osteopathic-manual* OR osteopathic-manip* OR osteopathic-treatment* OR osteopathic-thera*' OR cranial-osteopath* OR visceral-osteopath* OR 'osteopathic research' OR 'osteopathic care' OR osteopathic-intervention* OR reflexolog* OR 'applied kinesiology' OR soft-tissue-manip* OR musculoskeletal-manip* OR physical-therap* OR physiotherap* OR bodywork* OR manual-therap* OR manipulative-therap* OR manipulation-therap* OR 'myofascial structural integration' OR 'myofascial release' OR rolfing OR rolf OR fascia* OR fasciae OR myofascia*) | 128235 |
| 2 | (((TI=((oxytocin* OR cortisol OR neurotensin OR orexin OR cytokines OR neuropeptides) OR ((oxytocin* OR cortisol* OR hydrocortisone*) AND (biochemical* OR endocrine* OR neuroendocrine* OR neurohormone*)) )) OR AB=((oxytocin* OR cortisol OR neurotensin OR orexin OR cytokines OR neuropeptides) OR ((oxytocin* OR cortisol* OR hydrocortisone*) AND (biochemical* OR endocrine* OR neuroendocrine* OR neurohormone*)) )) OR AK=((oxytocin* OR cortisol OR neurotensin OR orexin OR cytokines OR neuropeptides) OR ((oxytocin* OR cortisol* OR hydrocortisone*) AND (biochemical* OR endocrine* OR neuroendocrine* OR neurohormone*)) )) OR KP=((oxytocin* OR cortisol OR neurotensin OR orexin OR cytokines OR neuropeptides) OR ((oxytocin* OR cortisol* OR hydrocortisone*) AND (biochemical* OR endocrine* OR neuroendocrine* OR neurohormone*)) ) | 544130 |
| 3 | (((TI=(human*)) OR AB=( human*)) OR AK=(human*)) OR KP=(human*) | 4571504 |
| 4 | #1 AND #2 AND #3 | 160 |

Notes: *Searched in Web of Science Core Collection, editions included: Science Citation Index Expanded (SCI-EXPANDED) 1990-present; Social Sciences Citation Index (SSCI) 1990-present; & Emerging Sources Citation Index (ESCI) 2018-present.*

**Supplementary Appendix 1.** Search strategy was developed from inception through July 2025, in collaboration with a research librarian (RB), and was applied across five databases: PubMed, CINAHL, Embase, Scopus, and Web of Science
